# Radiological assessment of shoulder balance following posterior spinal fusion for thoracic adolescent idiopathic scoliosis

**DOI:** 10.1186/1748-7161-10-S1-P29

**Published:** 2015-01-19

**Authors:** Takashi Namikawa, Akira Matsumura, Minori Kato, Kazunori Hayashi, Kenichi Kazuki, Hiroaki Nakamura

**Affiliations:** 1 Department of Orthopedic Surgery, Osaka City General Hospital, Japan; 2 Department of Orthopedic Surgery, Osaka City University, Japan

## Objective

The objective of the study was to evaluate shoulder balance following posterior spinal fusion for thoracic AIS.

## Methods

A total of 24 patients (22 female and 2 male) with thoracic AIS who had undergone posterior fusion with segmental pedicle screws were retrospectively reviewed. Proximal thoracic, main thoracic Cobb angle (PT and MT), % correction of both curve (PTC and MTC), T1 tilt, and shoulder asymmetry by the radiographic soft tissue shadow (RSH) were measured from preoperative, immediately postoperative and latest f/u postoperative radiographs. Preoperative PT and MT curve side-bending % correction were also measured (PTBC and MTBC). PTC:MTC ratio was defined as an index of PTC and MTC matching. The cases were divided into 2 groups from the immediately postoperative radiograph findings: Balanced group (RSH<20mm), Imbalanced group (RSH>=20mm). Preoperative RSH, PTBC, MTBC, PTC, MTC, pre- and postoperative T1 tilt, PTC:MTC ratio were compared between 2 groups.

## Results

A mean f/u period was 29 months (24-55). Fifteen patients had Lenke type 1 curve, 7 had type 2 curve and 2 had type 3 curve. A mean PT and MT were 33.0 degrees and 64.2 degrees before surgery, 16.1 degrees (50.5%) and 16.8 degrees (74.0%) immediately after surgery, and 16.9 degrees (49.0%) and 19.2 degrees (70.3%) at the latest f/u, respectively. A mean preoperative RSH of -12.3mm was changed to +11.1mm immediately after surgery and improved to +5.7mm at the latest f/u. Seventeen cases were "Balanced" and 7 cases were "Imbalanced" immediately after surgery. There were statistically significant differences in PTC (p=0.04), postoperative T1 tilt (p=0.04) and PTC:MTC ratio (p=0.02) between groups (Wilcoxon rank-sum test). There was only 1 patient whose shoulder had been remaining imbalanced at the latest f/u. She had marked shoulder imbalance immediately after surgery (RSH: +40mm).

## Conclusion

Sufficient PT curve correction what is matched to MT curve correction would be necessary to prevent postoperative shoulder imbalance. Almost cases in the series had satisfactory results in terms of shoulder balance at latest f/u, however, some cases those had marked shoulder imbalance immediately after surgery may have residual shoulder imbalance in the long term.

